# Chondromyxoid Fibroma of the Nasal Cavity: A Benign Tumor in a Rare Head and Neck Location

**DOI:** 10.7759/cureus.90488

**Published:** 2025-08-19

**Authors:** Chelsea Onyeji, Alycia Boutrand, Michael Ferguson

**Affiliations:** 1 Otolaryngology - Head and Neck Surgery, WakeMed, Raleigh, USA; 2 Otolaryngology - Head and Neck Surgery, University of North Carolina at Chapel Hill School of Medicine, Chapel Hill, USA

**Keywords:** bone tumor, chondromyxoid fibroma, head and neck, nasal mass, otolaryngology, primary bone neoplasms, rare nasal lesion

## Abstract

Chondromyxoid fibroma (CMF) is a rare benign bone tumor that infrequently involves the craniofacial skeleton. Its diagnosis can be challenging due to overlapping imaging features with other lesions. We report a case of a 72-year-old female in whom a right posterior nasal mass was incidentally identified on MRI. The patient was asymptomatic, and endoscopic examination revealed a spherical lesion near the posterior inferior turbinate. CMF was confirmed on CT imaging and histopathologic analysis. This case highlights an unusual presentation of CMF in the posterior nasal cavity. It also emphasizes the importance of a thorough workup, including imaging and histopathologic data, as CMF in the head and neck region often mimics more aggressive neoplasms, posing diagnostic challenges.

## Introduction

Chondromyxoid fibroma (CMF) is a rare benign bone tumor, accounting for less than 1% of all primary bone neoplasms [1]. It most commonly arises in the metaphyseal regions of long bones such as the tibia and femur [2]. Craniofacial involvement is exceedingly rare, occurring in approximately 5% of cases, most commonly in the mandible or maxilla, with isolated reports describing its presence in the sinonasal region [2].

When CMF involves the head and neck, it typically presents with nonspecific symptoms resulting from mass effect, or it may be discovered incidentally [2-4]. Imaging and histopathologic analysis are essential for distinguishing CMF from other entities and for guiding appropriate surgical management. Histologically, it is composed of stellate or spindle-shaped cells within a chondroid and myxoid stroma, often arranged in a multilobulated architecture [1]. On MRI, CMF can appear as a T1-hypointense or T2-hyperintense lesion, while on CT, bony erosion or remodeling can be seen [1]. Due to its rarity in this location, CMF is seldom included in the initial differential diagnosis of nasal masses, as its imaging characteristics may resemble those of other benign or malignant lesions. Here, we report an unusual presentation of CMF in the posterior nasal cavity in a 72-year-old female. This case highlights the role of radiologic and pathologic correlation in achieving an accurate diagnosis.

## Case presentation

**Figure 1:**
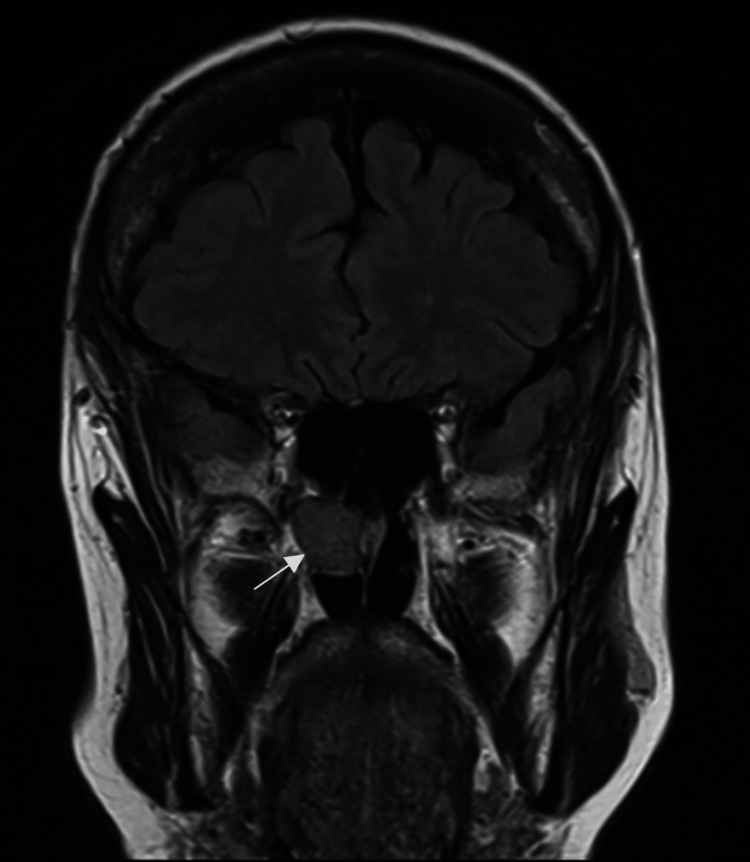
T1-weighted brain MRI without contrast. A hypointense lesion measuring approximately 16 mm, located along the posterior margin of the right nasal cavity.

A 72-year-old female was referred to our otolaryngology clinic after a right nasal mass was incidentally discovered on an MRI that was done for unrelated reasons. Non-contrast T1-weighted MRI had revealed a lesion along the posterior margin of the right nasal cavity (Figure 1). This lesion was indeterminate in nature, but it suggested fibrous dysplasia.

On evaluation, the patient denied symptoms such as nasal obstruction, hoarseness, dysphagia, or dyspnea. Physical examination showed no facial tenderness or sinus pain. Nasal endoscopy revealed a spherical, non-ulcerated lesion in the posterior nasal cavity contiguous with the posterior portion of the inferior turbinate. 

**Figure 2:**
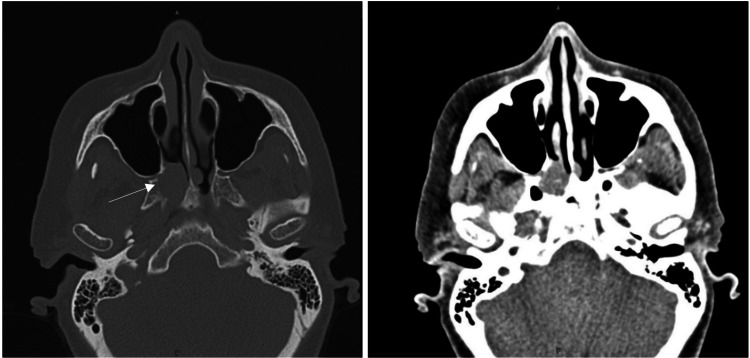
Non-contrast CT of the paranasal sinuses. Soft tissue mass centered at the right posterior nasal aperture (choana) with associated osseous scalloping and erosion of the medial margin of the right medial pterygoid plate.

Before resection, a computed tomography (CT) scan was done to further characterize the mass and plan surgical intervention. The CT confirmed the presence of a 2.2 cm lesion in the right choana, associated with osseous remodeling and erosion (Figure 2). Given these findings, a neoplastic process could not be excluded. 

**Figure 3:**
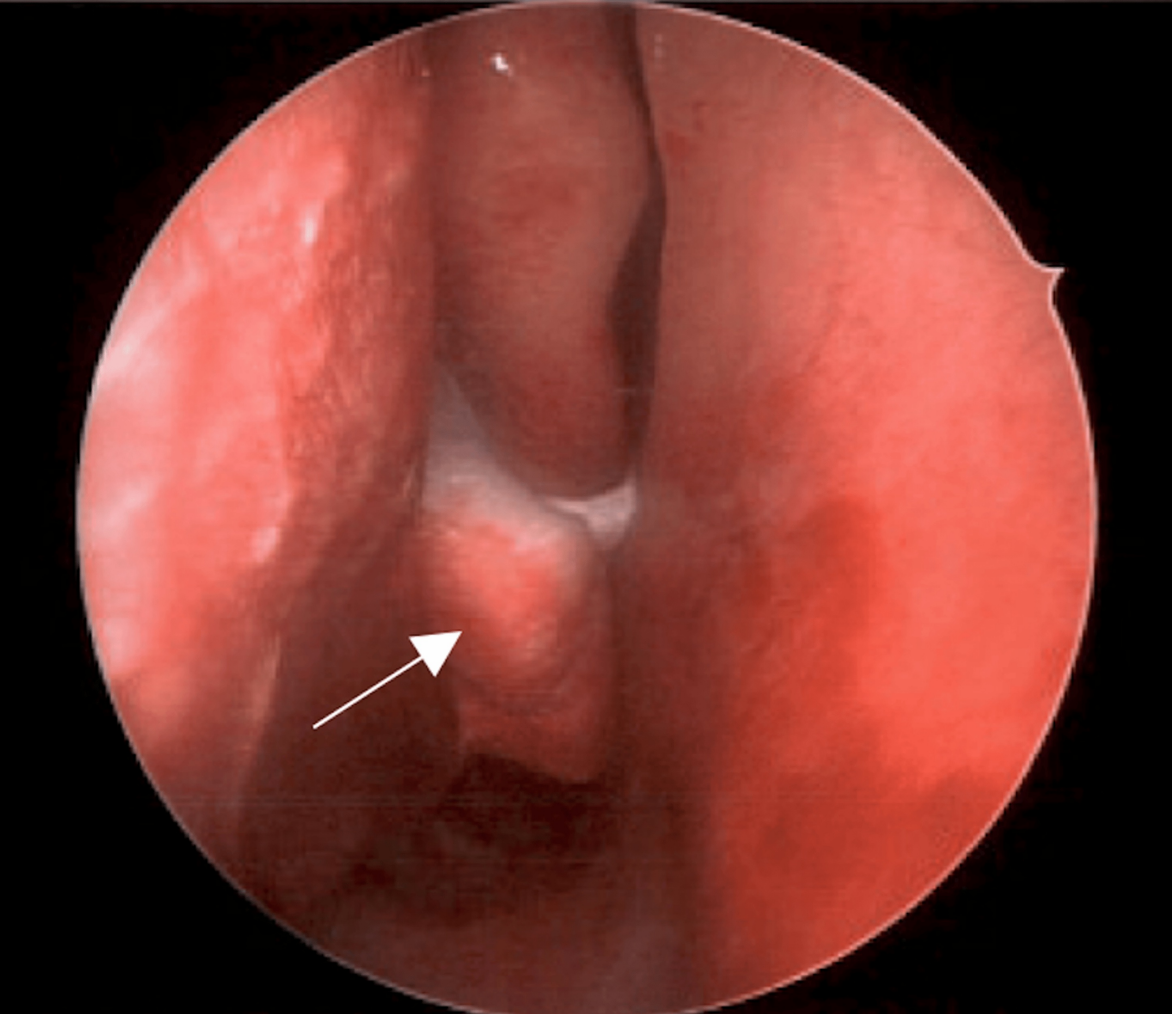
Intraoperative photo. Endoscopic view demonstrating a mass located inferior to the middle turbinate of the right nasal cavity. It was well-circumscribed in appearance, with a smooth, vascular mucosal covering.

**Figure 4:**
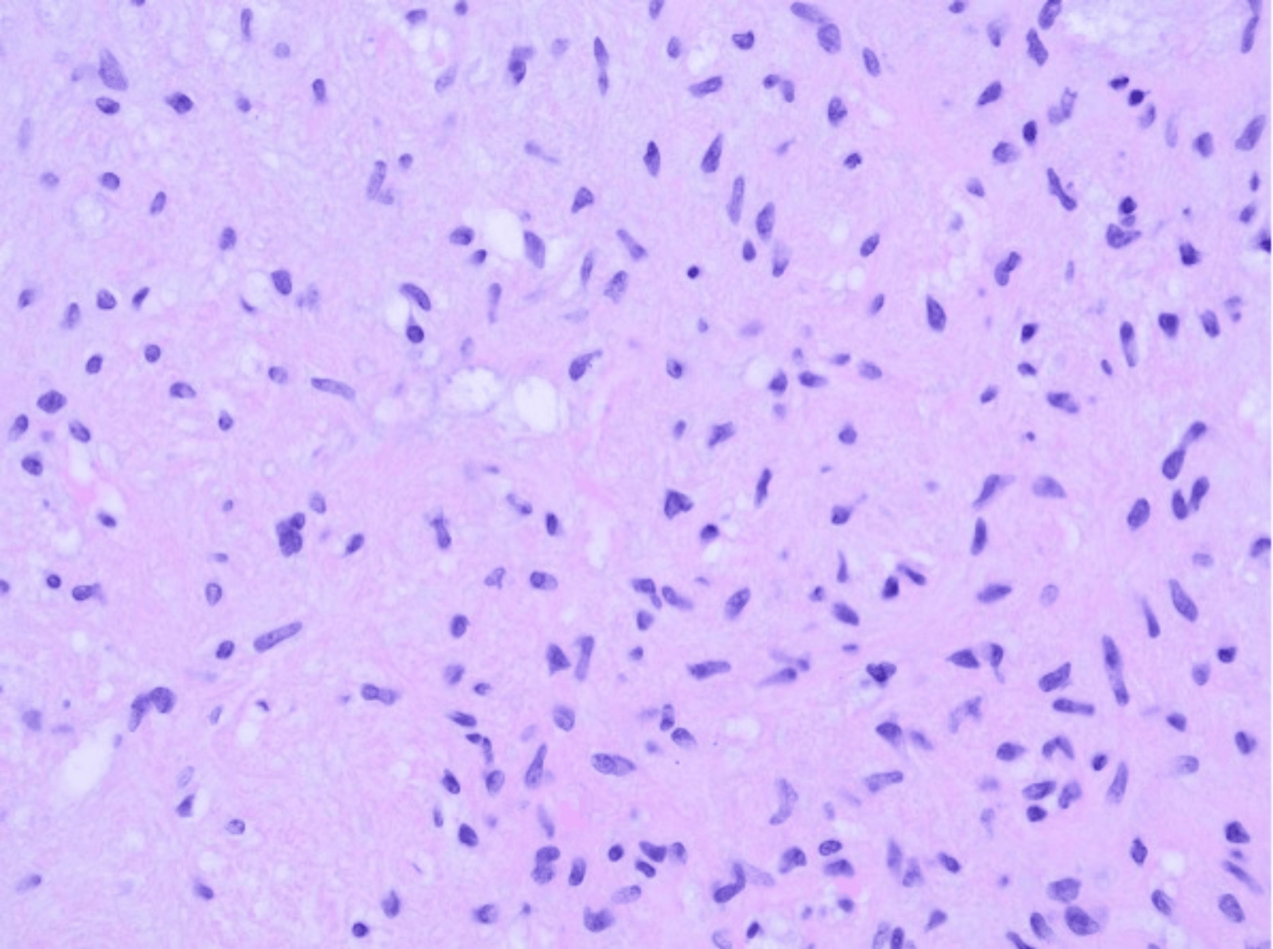
Histopathologic images of the resected nasal mass at 400x magnification. There is a prominent myxoid stroma containing uniform stellate and spindle cells. Hypocellular regions contain foci of immature chondroid matrix.

The patient underwent endoscopic resection under general anesthesia. The mass was located below the middle turbinate and appeared well-circumscribed (Figure 3). After mucosal debridement, a firm, white mass was exposed, raising suspicion for a neoplastic process. Histopathological examination revealed multilobulated tissue with prominent myxoid stroma populated by uniform stellate and spindle cells and early chondroid matrix. (Figure 4). Immunohistochemical staining showed focal positivity for smooth muscle actin. These features were consistent with a diagnosis of chondromyxoid fibroma. 

The patient was discharged 24 hours postoperatively without complications. Given the rare occurrence of CMF in the craniofacial skeleton and the possibility of recurrence with incomplete excision, she will undergo continued surveillance.

## Discussion

Chondromyxoid fibroma is a rare benign bone tumor, representing less than 1% of all primary bone tumors, and it typically arises in the metaphyseal regions of long bones [1,5]. Craniofacial involvement is exceedingly uncommon, with only isolated reports describing CMF in the nasal cavity, septum, maxilla, or skull base [2,6]. Due to its rarity in the sinonasal region, CMF is often not initially considered in the differential diagnosis, and its imaging characteristics can mimic those of other benign or malignant lesions [1,7-8]

When CMF occurs in the head and neck, it generally presents with nonspecific symptoms secondary to mass effect, including facial pain, nasal congestion, epistaxis, or persistent rhinosinusitis. [2-4] Interestingly, in our case, the lesion was incidentally discovered on MRI in an asymptomatic patient. Given that the radiologic features of CMF often resemble other cartilaginous tumors such as chondrosarcoma and fibrous dysplasia, comprehensive imaging evaluation and histopathologic analysis are essential for establishing an accurate diagnosis and planning appropriate surgical management [4,8].

MRI is preferred for assessing soft tissue involvement in sinonasal masses, whereas CT is more effective for characterizing bony erosion, thinning, or remodeling [1,3]. In this case, CT imaging demonstrated a lesion with osseous scalloping and cortical erosion consistent with CMF. However, the lesion was initially interpreted as fibrous dysplasia, likely due to its location and similar sclerotic radiographic appearance. Chondrosarcoma, another important consideration, typically appears poorly circumscribed with prominent calcifications, in contrast to the microscopic calcifications of CMF, which are rarely visible on imaging. [1,3,8]. These overlapping radiographic features highlight the necessity of histopathologic confirmation, particularly for lesions involving the skull and facial bones, where neoplasia is a significant concern.

Histopathologic examination in this case revealed a grossly firm, multilobulated mass. Microscopically, it demonstrated alternating areas of hypo- and hypercellularity with prominent myxoid stroma, classic features seen in CMF. Immunohistochemistry generally has limited diagnostic utility in CMF, as some markers may be shared with other cartilaginous tumors [4]. CMF may show positivity for S100, depending on the degree of chondroblastic differentiation, as well as smooth muscle actin and CD34, reflecting its myofibroblastic features [1].

Surgical excision remains the primary treatment for craniofacial CMF, with en bloc resection preferred to minimize recurrence and the potential risk of malignant transformation [1,3]. However, achieving complete resection can be challenging due to the proximity to critical structures and concerns for cosmetic or functional compromise. Consequently, some surgeons advocate for curettage with close follow-up in select sinonasal cases [3], although this approach carries a recurrence rate of up to 17% [1,4]. A systematic review by De La Peña et al. confirmed that gross total resection is associated with lower recurrence rates, reinforcing the importance of adequate excision [9].

While radiation therapy is generally avoided due to the theoretical risk of malignant transformation, it may be considered when complete resection is not feasible [3,10]. In our case, complete endoscopic resection was achieved without complication. Nevertheless, given the risk of recurrence associated with sinonasal CMF, ongoing MRI surveillance remains essential.

## Conclusions

The incidental discovery of an asymptomatic nasal mass in this patient illustrates how CMF may present without overt clinical signs, making radiologic and histopathologic correlation critical for accurate diagnosis. The sclerotic radiographic appearance of CMF can be suggestive of other neoplasms such as fibrous dysplasia or chondrosarcoma. This lack of specificity emphasizes the definitive role of histopathology in distinguishing CMF from malignant or other benign lesions.

Surgical resection remains the mainstay of treatment, and complete excision offers the best chance of minimizing recurrence. Due to the tumor’s potential for regrowth, especially in anatomically complex areas, long-term radiologic surveillance is essential. This case contributes to the limited literature on sinonasal CMF and highlights the diagnostic and management challenges posed by such rare presentations.
